# Cardiac Arrhythmia Following an Epileptic Seizure

**DOI:** 10.5811/cpcem.2019.6.43173

**Published:** 2019-08-05

**Authors:** Hani I. Kuttab, Elizabeth A. Harris, Katie L. Tataris, James Tao, David G. Beiser

**Affiliations:** University of Chicago Medical Center, Section of Emergency Medicine, Chicago, Illinois

## Abstract

Sudden unexplained death in epilepsy (SUDEP) refers to a death in a patient with epilepsy that is not due to trauma, drowning, status epilepticus, or another apparent cause. Although the pathophysiology of SUDEP is incompletely understood, growing evidence supports the role of seizure-associated arrhythmias as a potential etiology. We present a unique case of a patient presenting with ventricular tachycardia shortly following a seizure, along with corresponding laboratory data. Awareness of high risk arrhythmias in seizure patients could lead to advances in understanding pathophysiology and treatment of this complication of seizure disorder and ultimately prevention of SUDEP.

## INTRODUCTION

Sudden unexpected death in epilepsy (SUDEP) refers to death in a patient with epilepsy that is not due to trauma, drowning, status epilepticus, or another known cause for which there is often evidence of an associated seizure. It has been estimated that SUDEP accounts for approximately 10% of deaths in patients with seizure disorder each year in the United States, and accounts for 20–30% of epilepsy-related deaths, although this may differ across study populations.^1^–^2^ Typically the patient is found dead, and the diagnosis is confirmed only when clinical criteria are met and autopsy reveals no alternative cause of death.^1^–^2^ The etiology of SUDEP is uncertain, although observations in individual cases suggest possible cardiogenic, pulmonary, and/or primary neurologic pathophysiologic mechanisms.^3^–^4^ We present a case of a patient presenting with ventricular tachycardia immediately following a seizure, along with corresponding laboratory data.

## CASE REPORT

A 17-year-old male with a history of cognitive impairment and epilepsy presented via emergency medical services (EMS) to the emergency department (ED) following a witnessed seizure. Upon arrival, the patient was noted to be postictal; thus, history was obtained from the patient’s mother who reported that he had experienced symptoms consistent with a generalized tonic-clonic seizure earlier in the day. It was unknown if the patient returned to baseline; however, the mother offered that following this seizure, he did state that he needed a refill of his home levetiracetam. She called EMS for transport to the ED. Upon EMS arrival, the patient suffered another seizure, described by EMS as generalized tonic-clonic activity lasting approximately three minutes. The patient was found sitting on the couch and subsequently fell forward onto the ground. EMS administered five milligrams (mg) of intramuscular midazolam and then established intravenous (IV) access. He had no further seizure-like activity during transport or upon arrival to the ED.

**Image 1:**
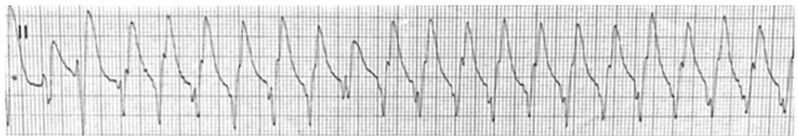
Rhythm strip demonstrating ventricular tachycardia (monomorphic). Heart rate = 143 beats per minute.

**Image 2:**
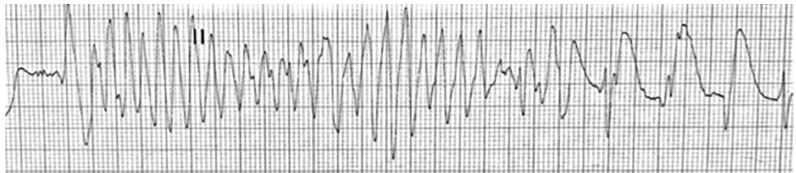
Rhythm strip demonstrating ventricular tachycardia (polymorphic). Heart rate = 172 beats per minute.

An electrocardiogram obtained during transport documented a monomorphic ventricular tachycardia (Image 1). Shortly thereafter, this rhythm degenerated into a polymorphic ventricular tachycardia (Image 2).

The patient arrived to the ED in normal sinus rhythm. During transport, he was not witnessed to have seizure activity, nor did he lose pulses. No further medications or interventions were administered or performed, and no additional arrhythmias were noted during hospitalization.

Upon arrival to the ED, the patient was somnolent, moving all extremities spontaneously, and responsive only to pain. A point-of-care venous blood gas was obtained and significant for a pH of 6.78 (normal 7.35–7.38), carbon dioxide (CO_2_) 100 millimeters of mercury (mmHg) (normal 44–48 mmHg), a normal blood glucose, and lactate of 13.8 mg per deciliter (dL) (normal <2.0 mg/dL). The patient received two liters of IV fluids and a two-gram IV loading dose of levetiracetam. The patient was also placed on supplemental oxygen by nasal cannula. Subsequent blood gases thereafter showed improvement in both metabolic and respiratory acidosis (pH 7.0, CO_2_ 62 mmHg, lactate 10 mg/dL). The patient’s high-sensitivity troponin T resulted at 22 nanogram per liter (ng/L) (normal <14 ng/L). The magnesium, potassium, and calcium levels were normal. Electrocardiogram obtained in the ED was significant for sinus tachycardia and a corrected QT interval of 398 milliseconds.

The patient was admitted to the pediatric intensive care unit and underwent correction of all metabolic abnormalities. Later, the serum levetiracetam resulted at <2.0 micrograms per milliliter (ug/mL) (therapeutic levels 12.0–46.0 ug/mL) and the valproic acid level at 112 ug/mL (therapeutic levels 50–125 ug/mL). The patient was seen by the pediatric cardiology team, had a normal pediatric echocardiogram, and was discharged home with a 30-day cardiac monitoring device, ultimately demonstrating no additional arrhythmias.

## DISCUSSION

Meta-analysis of peri-ictal cardiac arrhythmias reveals that ictal asystole, ictal bradycardia, and postictal atrial flutter/fibrillation are the most common presenting arrhythmias related to seizure, and are often self-limiting.^4^ Proposed mechanisms include direct stimulation of the central autonomic network (i.e., cingulated gyrus, amygdala, or insular cortex) and seizure-induced catecholamine release leading to vasovagal responses.^3^ Both peri-ictal ventricular tachycardia and fibrillation have been described in epilepsy patients with no underlying cardiac disease.^5^ It has also been suggested that pathologic cardiac repolarization (including QT prolongation, QT shortening, and increased dispersion) is responsible for tachyarrthymias in these patients, and ultimately leads to SUDEP.^5^ Recent data also suggests a significant association between potentially high risk cardiac arrhythmias and the duration of ictal/postictal oxygen desaturation.^6^ Ultimately, cerebral anoxia from asystole ceases seizure activity.

**CPC-EM Capsule**

What do we already know about this clinical entity?

*Sudden, Unexplained Death in Epilepsy (SUDEP) accounts for death in 20–30% of patients with epilepsy. However, the pathophysiology is not well understood*.

What makes this presentation of disease reportable?

*Pre-hospital rhythm strips captured a cardiac arrhythmia in a patient following a seizure, suggesting that SUDEP may be caused by a cardiac arrhythmia*.

What is the major learning point?

*Emergency department providers must be aware of cardiac arrhythmias or near-SUDEP in patients immediately following a seizure*.

How might this improve emergency medicine practice?

*With better understanding of SUDEP and cardiac arrhythmias in post-ictal patients, providers can take measures to prevent apnea and correct subsequent metabolic derangements*.

The above case describes a patient with postictal ventricular tachycardia in the setting of markedly deranged blood gas values, pointing to respiratory dysfunction and subsequent metabolic and respiratory acidosis as an additional plausible etiology of SUDEP. However, cardiac arrhythmias sometimes coincide with epileptic seizures, and some individuals may have components of both cardiogenic syncope and epilepsy. In a recent case report of a patient presenting to an ED with a seizure, automatic implanted cardioverter defibrillator interrogation revealed ventricular tachycardia and fibrillation.^8^ In our case, this patient had no recurrence of cardiac arrhythmias following interventions as described above, and fortunately did not succumb to SUDEP.

In the seizing patient, measures must be taken to avoid apnea and correct hypoxemia and metabolic derangements. ED providers must be aware of cardiac arrhythmias or near-SUDEP following a seizure. Early nursing interventions, including administration of supplemental oxygen, oropharyngeal suctioning, and patient repositioning have been shown to reduce the duration of respiratory-induced hypoxemia.^7^ Providers should also be aware of the use of benzodiazepines as a potential cause of worsening respiratory depression and subsequent respiratory acidosis, leading to the development of arrhythmias. Finally, in postictal patients, physicians should consider electrocardiograms and continuous telemetry monitoring while rapidly reversing metabolic derangements.

## CONCLUSION

More knowledge about the cardiovascular status of epileptic patients during, between, and immediately after seizures is needed to better understand and prevent high-risk arrhythmias and SUDEP by measures such as cardioprotective drugs, respiratory therapy, or implantation of a defibrillator.^3^ Specifically, more knowledge and awareness of this phenomenon in the emergency medicine community is necessary to best care for these patients in the acute setting.
